# CDK4/6 Inhibitors in Advanced HR+/HER2 − Breast Cancer: A Multicenter Real-World Data Analysis

**DOI:** 10.1159/000527917

**Published:** 2022-12-06

**Authors:** Carolin Müller, Verena Kiver, Erich-Franz Solomayer, Gudrun Wagenpfeil, Caroline Neeb, Jens-Uwe Blohmer, Alina Valik Abramian, Nicolai Maass, Florian Schütz, Cornelia Kolberg-Liedtke, Damian Johannes Ralser, Anna-Christina Rambow

**Affiliations:** ^a^Department of Gynecology, Obstetrics and Reproductive Medicine, Saarland University Medical Center, Homburg, Germany; ^b^Department of Gynecology with Breast Center, Charité-Universitätsmedizin Berlin, Corporate Member of Freie Universität Berlin and Humboldt-Universität zu Berlin, Berlin, Germany; ^c^Institute for Medical Biometry, Epidemiology and Medical Informatics (IMBEI), Saarland University Medical Center, Homburg, Germany; ^d^Department of Gynecology and Obstetrics, University Medical Center Bonn, Bonn, Germany; ^e^Department of Gynecology and Obstetrics, University Medical Center Schleswig-Holstein (UKSH), Kiel, Germany; ^f^Department of Gynecology and Obstetrics, Diakonissen-Stiftungs-Krankenhaus Speyer, Speyer, Germany; ^g^Department of Gynecology and Obstetrics, University Medical Center Essen, Essen, Germany

**Keywords:** CDK4/6 inhibitors, Metastatic breast cancer, Progression-free survival, Real-world data

## Abstract

**Purpose:**

CDK4/6 inhibitors (CDK4/6i) combined with endocrine therapy are considered standard-of-care for first-line therapy of patients with hormone receptor positive, HER2 negative, advanced breast cancer (HR+/HER2− ABC). Superiority of combination therapy over endocrine monotherapy has been demonstrated in a multitude of randomized controlled trials (RCTs) in phase III and IV. However, RCTs reflect clinical reality only to a limited extent, as narrow inclusion criteria lead to a selected patient collective. Here, we present real-world data (RWD) on CDK4/6i treatment in patients with HR+/HER2− ABC at four certified German university breast cancer centers.

**Methods:**

Patients diagnosed with HR+/HER2− ABC who were treated in clinical routine with CDK4/6i between November 2016 and December 2020 at four certified German university breast cancer centers (Saarland University Medical Center, University Medical Center Charité Berlin, University Medical Center Bonn, and University Medical Center Hospital Schleswig-Holstein, Campus Kiel) were identified and enrolled in this retrospective study. Clinicopathological characteristics and clinical outcomes were recorded with particular emphasis on CDK4/6i therapy course [progression-free survival (PFS) following treatment initiation, toxicity, dose reduction, therapy discontinuation, prior and subsequent therapy line].

**Results:**

Data from *n* = 448 patients were evaluated. The mean patient age was 63 (±12) years. Of these patients, *n* = 165 (36.8%) were primarily metastasized, and *n* = 283 (63.2%) had secondary metastatic disease. *N* = 319 patients (71.3%) received palbociclib, *n* = 114 patients (25.4%) received ribociclib, and *n* = 15 patients (3.3%) received abemaciclib, respectively. Dose reduction was performed in *n* = 132 cases (29.5%). *N* = 57 patients (12.7%) discontinued the treatment with CDK4/6i due to side effects. *N* = 196 patients (43.8%) experienced disease progression under CDK4/6i treatment. The median PFS was 17 months. Presence of hepatic metastases and prior therapy lines were associated with shorter PFS, whereas estrogen positivity and dose reduction due to toxicity were positively associated with PFS. Presence of bone and lung metastases, progesterone positivity, Ki67 index, grading, *BRCA1/2* and *PIK3CA* mutation status, adjuvant endocrine resistance, and age did not significantly impact on PFS.

**Conclusion:**

Our RWD analysis on CDK4/6i treatment in Germany supports data from RCTs regarding both treatment efficacy and safety of CDK4/6i for treatment of patients with HR+/HER2− ABC. In comparison to data from the pivotal RCTs, median PFS was lower but within the expected range for RWD, which could result from inclusion of patients with more advanced diseases (i.e., higher therapy lines) to our dataset.

## Introduction

Implementation of CDK4/6 inhibitors (CDK4/6i) into clinical routine has revolutionized the treatment of patients with hormone receptor positive, HER2 negative advanced breast cancer (HR+/HER2− ABC). Addition of CDK4/6i to endocrine therapy (ET) has demonstrated superiority in several randomized controlled trials (RCTs) in phase III and IV with an increased progression-free survival (PFS) compared to ET monotherapy across various patient subgroups including patients with de novo and recurrent metastatic disease, pre- and postmenopausal women, progesterone receptor negative disease, bone- and visceral-metastases. Several RCTs, namely PALOMA-3 for palbociclib [1], MONALEESA-3 for ribociclib [2, 3] and MONARCH-2 for abemaciclib [4, 5] led to approval of CDK4/6i plus ET as first-line treatment for HR+/HER2− ABC. However, these RCTs reflect clinical reality only to a limited extent, as narrow inclusion criteria lead to a selected patient collective. Especially elderly patients, patients with comorbidities or treatment in further therapy lines, are underrepresented in these RCTs. In this context, the analysis of “real-world data” (RWD) has become increasingly important in recent years. RWD is consulted to assess treatment effectiveness, tolerability and to reproduce evidence of clinical benefit and safety of RCT results [6, 7]. Currently, several studies are collecting RWD with regard to CDK4/6i: the PERFORM study [8] aims to report clinical and scientific data as well as patient reported outcomes of palbociclib in combination with ET as first-line treatment in HR+/HER2− ABC. Results are pending. However, this study will not provide any information regarding CDK4/6i administered in second, third, or further therapy lines. Further, only patients receiving palbociclib are included in the study thereby representing only one of three available CDK4/6i. The IRIS trial retrospectively collected RWD from patients all over the world, demonstrating an improved PFS [9, 10]. However, this study also only included patients who received palbociclib. There are two noninterventional observational studies evaluating the other two CDK4/6i, namely the Ribanna trial for ribociclib (NCT02941926) and the IMPACT trial for abemaciclib (NCT04352777). Results have not yet been published. Furthermore, the German network “PRAEGNANT” prospectively collects data of patients with ABC to perform translational research and to optimize oncological therapy [11].

To the best of our knowledge, there are no RWD available regarding the use of all three CDK4/6i independent from the line of therapy. Here, we aimed to analyze the use of CDK4/6i and clinical outcome of patients with HR+/HER2− ABC at four certified German university breast cancer centers in clinical routine.

## Patients and Methods

### Data Collection

Patients diagnosed with HR+/HER2-locally advanced or metastatic breast cancer who were treated with CDK4/6i in clinical routine at four certified German university breast cancer centers (Saarland University Medical Center, University Medical Center Charité Berlin, University Medical Center Bonn and University Medical Center Schleswig-Holstein, Campus Kiel) were identified and enrolled in this retrospective study. Data collection spans the time from approval of the first CDK4/6i palbociclib in Germany in November 2016 to December 2020. Exclusion criterion was participation in an interventional pivotal study.

Patient characteristics were collected from routine clinical documentation. Extracted data included age, tumor biology (receptor status, Ki67 index, grading), de novo metastatic disease or recurrent disease, localization of metastatic disease, *BRCA1/2* and *PIK3CA* mutation status, and date of death. Emphasis was put on CDK4/6i therapy course (PFS following treatment initiation, toxicity, dose reduction, therapy discontinuation, prior and subsequent therapy line).

### Statistics

Statistical analyses were performed using SPSS 28.0 (IBM, Armonk, USA). Quantitative parameters (e.g., patients' age) are given as mean with standard deviation. Qualitative parameters (e.g., tumor stage) are presented as frequencies. Kaplan-Meier analysis and Cox regression were performed to analyze PFS and confounders on PFS. Variables influencing the discontinuation of therapy due to side effects were determined with regression analysis.

## Results

In total, data from *n* = 448 patients who were treated with CDK4/6i at four certified German university breast cancer centers between November 2016 and December 2020 were included in the study. The patient mean age was 63 (±12) years. Of these patients, *n* = 165 (36.8%) were primarily metastasized, and *n* = 283 (63.2%) had secondary metastatic disease. *N* = 334 patients (74.6%) had osseous metastases, *n* = 154 patients (34.4%) had pulmonary metastases, *n* = 133 patients (29.7%) had hepatic metastases, and *n* = 219 patients (48.9%) had exhibited other sites of metastases (e.g., lymphatic, pleural, cutaneous, peritoneal, cerebral). Tumor characteristics including tumor stage and tumor biology are shown in Table 1. Tumor characteristics are given for metastatic disease as well as for primary breast cancer if available. Interestingly, *n* = 14 patients (3.1%) showed a receptor discordance from initially triple negative or HER2 positive nonmetastatic BC to HR+/HER2− ABC. Of the *n* = 283 patients with secondary metastasis, *n* = 69 (15.4%) patients had no HR status determined at the metastatic disease stage. Of these patients with unknown HR status, *n* = 23 (5.1%) displayed primary resistance to CDK4/6i treatment that was defined as disease progression within the first 6 months of treatment in metastatic setting. Of all patients, a total of *n* = 48 patients suffered from primary endocrine resistance in the metastatic setting (defined as disease progression within the first 6 months of first-line CDK4/6i treatment).

*n* = 85 patients (19.0%) were tested for *PIK3CA* mutations. In *n* = 21 of these patients (4.7% of all patients), a *PIK3CA* mutation was detected. *BRCA1/2* mutation status was tested in *n* = 91 patients (20.3%) with identification of 8 pathogenic mutations (1.8% of all patients).

In our cohort, *n* = 319 patients (71.3%) received palbociclib, *n* = 114 patients (25.4%) received ribociclib, and *n* = 15 patients (3.3%) received abemaciclib, respectively. The selection of the CDK4/6i was independent of patient's age (mean age: palbociclib 63 ± 11,7, ribociclib 62 ± 12.7, abemaciclib 62 ± 12.2 years, respectively). Given the unequal group sizes and the different follow-up periods due to the different approval dates, we did not distinguish between the three CDK4/6i. Table 2 shows in which therapy line patients received CDK4/6i. As endocrine partner, *n* = 291 patients (65.0%) received aromatase inhibitors and *n* = 156 patients (34.8%) Fulvestrant. *N* = 35 (7.8%) premenopausal patients were additionally treated with GnRH agonists. A total of *n* = 290 patients (64.7%) received concomitant anti-resorptive therapy, *n* = 13 patients (2.9%) received bisphosphonates, and *n* = 277 patients (61.8%) were treated with Denosumab, respectively.

Frequencies of dose reduction and discontinuation of treatment due to side effects are documented in Table 3. Overall, dose reduction was performed in *n* = 130 cases (29.0%). The most common reason for dose reduction [>65% of cases of dose reduction (*n* = 86)] was hematotoxicity; particularly, neutropenia CTCAE grade III or higher. Another common reason for dose reduction was a deterioration of general condition due to excessive side effects (e.g., nausea, fatigue; 2.5%). In 2.2% of cases, therapy was directly initiated at reduced doses due to known comorbidities or concomitant medication. *N* = 59 patients (13.2%) discontinued treatment with CDK4/6i due to side effects. Again, hematotoxicity (3.1%) and deterioration of general condition (2.9%) were the most common reasons for therapy discontinuation. Regarding medication, 10.0% of the patients (*n =* 32 patients) receiving palbociclib discontinued treatment because of side effects, whereas 19.3% (*n =* 22 patients) in the ribociclib group and 20.0% (*n =* 3 patients) in the abemaciclib group discontinued therapy due to toxicity. Possible confounders on treatment discontinuation due to side effects were analyzed using binary logistic regression. Patient age was the only parameter showing statistically significant influence (*p* = 0.012). If age increases by 1 year, the risk of discontinuing therapy due to side effects increases by 3.3% (odds ratio 1.033). All other tested factors (de novo vs. recurrent metastases, estrogen positivity, progesterone positivity, Ki67 index, grading, localization of metastases, and line of therapy) showed no influence on probability of treatment discontinuation with CDK4/6i due to side effects.

*N* = 196 patients (43.8%) discontinued CDK4/6i treatment due to disease progression. The site of progression is shown in Table 4. Most patients had a hepatic progression or newly emerged hepatic metastasis (*n* = 82, 18.3%). Previous and subsequent therapies are depicted in Table 5. In the metastatic setting, *n* = 169 patients (37.7%) were already pretreated prior initiation of CDK4/6i therapy. The majority of these patients had prior ET (*n* = 93, 20.8%). *N* = 20 patients (4.5%) received chemotherapy, whereas *n* = 56 patients (12.5%) received both, endocrine and chemotherapy before CDK4/6i therapy. After progression following CDK4/6i treatment, endocrine-based treatment was continued in *n* = 80 patients (17.9%). Almost half of these patients (*n* = 34; 7.6%) received everolimus in combination with exemestane. In regression analysis, subsequent treatment of everolimus and exemestane did not correlate with therapy line of CDK4/6i therapy (*p* = 0.69). A total of *n* = 50 patients received medication as part of a noninterventional study [Ribanna (NCT02941926), PRECYCLE (NCT03220178)].

In total, 197 patients (43.7%) continued CDK4/6i treatment over the observation period or until last follow-up. Overall, 92 patients (20.5%) died during the observation period, and 92 patients (20.5%) were lost to follow-up.

**Fig. 1:**
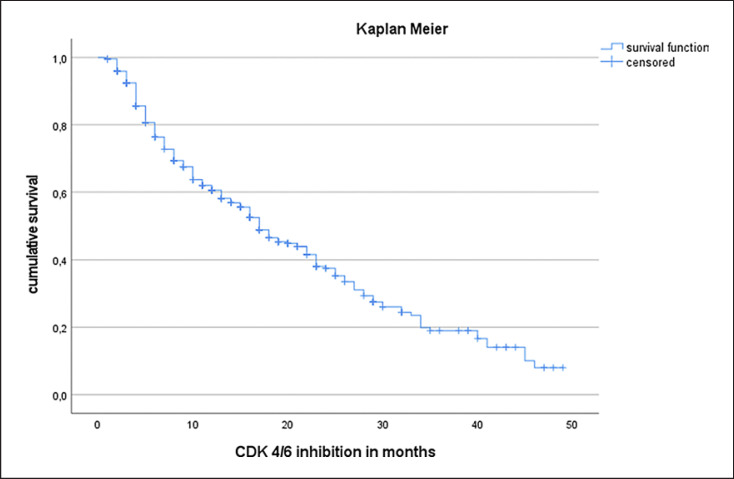
Progression-free survival in Kaplan-Meier analysis following CDK4/6 inhibitor treatment (median PFS 17 months).

**Fig. 2:**
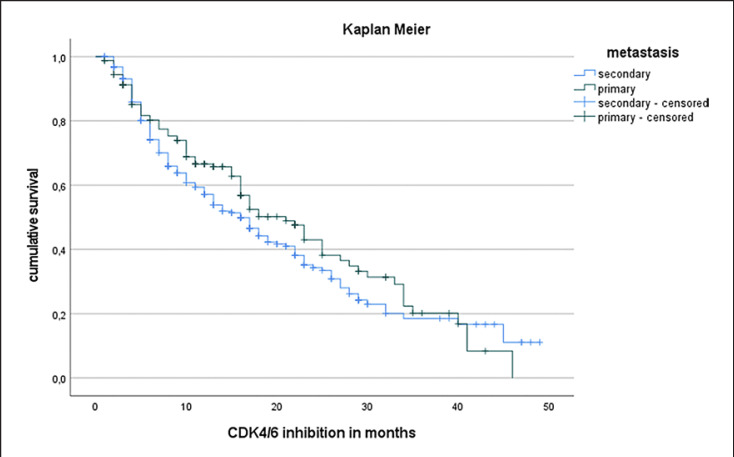
PFS in primary versus secondary metastatic patients in Kaplan-Meier analysis. Patients with primary metastasis showed a better median PFS (21 months) in comparison to secondary metastatic disease (median PFS 16 months) but without reaching statistical significance (Log-Rank test, *p* = 0.21).

**Fig. 3:**
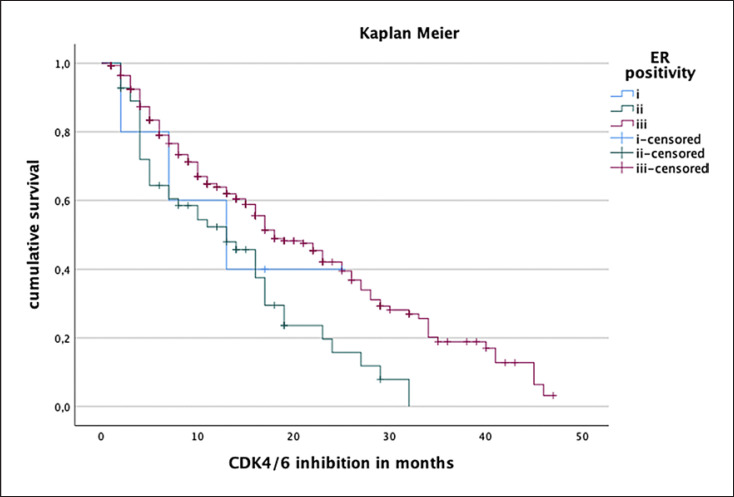
Influence of estrogen positivity on PFS in Kaplan-Meier analysis. Group (i): IRS 1–2 or IHC 1–10%. Group (ii): IRS 3–8 or 11–70%. Group (iii): IRS 9–12 or IHC 71–100%. Group (iii) showed a better PFS (18 months) compared to group (i) and (ii) (13 months) with log-rank test: *p* < 0.001.

The median PFS in the entire study cohort was 17 months (see Fig. 1). PFS was independent of patients' age (Cox regression: *p* = 0.176, hazard ratio 0.993). Patients with de novo metastasis showed a prolonged median PFS (21 months) compared to patients suffering from secondary metastatic disease (median PFS 16 months). Of note, this difference was not statistically significant (log-rank test, *p* = 0.21; Fig. 2). Regarding tumor biology, estrogen positivity showed significant influence on PFS. Estrogen positivity was further subgrouped: (1) immunoreactive score (IRS) 1–2 or immunohistochemistry score (IHC) 1–10%, (2) IRS 3–8 or IHC 11–70%, and (3) IRS 9–12 or IHC 71–100%. The third group with high estrogen receptor (ER) positivity showed an improved PFS (18 months) compared to the other two groups (13 months) with log-rank test: *p* < 0.001 (Fig. 3). In terms of progesterone positivity, a similar distinction was applied: (i) IRS 0 or IHC 0%, (ii) IRS 1–2 or IHC 1–10%, (iii) IRS 3–8 or 11–70%, and (iv) IRS 9–12 or IHC 71–100%. Progesterone positivity showed significant influence on PFS in univariate Kaplan-Meier analysis between (iii) and (i)/(ii) (*p* = 0.008/*p* = 0.022). However, this influence could not be confirmed in multivariate analysis. Patients with higher grading had a shorter PFS without reaching statistical significance (*p* = 0.205). A higher Ki67 index was associated with unfavorable PFS in univariate regression analysis (hazard ratio of 1.012; *p* = 0.003).

Further, we evaluated whether *BRCA1/2* and *PIK3CA* mutation status had an impact on PFS. Patients carrying a pathogenic *PIK3CA* mutation had a median PFS of 23 months compared to 15 months of patients without a *PIK3CA* mutation. However, this PFS difference showed no statistical significance (*p* = 0.26). *BRCA1/2* mutation carrier exhibited a median PFS of 27 months compared to 14 months of patients with negatively tested *BRCA1/2* mutation status. This PFS difference was not statistically significant (*p* = 0.12).

Next, we examined the extent to which the metastatic site affected PFS. Bone metastases did not affect the median PFS (log-rank test *p* = 0.176), whereas patients suffering from pulmonary or hepatic metastases had a shorter median PFS (pulmonary metastases 11 vs. 22 months; hepatic metastases 9 vs. 22 months; log-rank test *p* < 0.001). However, only hepatic metastases showed prognostic values regarding PFS in multivariate analysis.

Furthermore, we investigated whether the line of therapy in which CDK4/6i was administered had an impact on PFS. Median PFS following CDK4/6i in first-line treatment in the metastatic setting was 23 months compared to 13 months for second-line therapy and 11 months for further-line-treated patients (log-rank test *p* < 0.001). Concerning prior therapy for metastatic disease, the median PFS of patients who received chemotherapy prior to CDK4/6i was 9 months. Patients who only received ET monotherapy prior to CDK4/6i therapy initiation had a median PFS of 13 months under CDK4/6i. This difference showed no statistical difference (log-rank test, *p* = 0.385).

In addition, we examined the impact of adjuvant chemotherapy on CDK4/6i therapy course. In our RWD analysis, *n =* 194 patients received adjuvant chemotherapy, whereas *n =* 71 patients did not receive chemotherapy prior to secondary metastatic disease. PFS of patients who received adjuvant chemotherapy was 17 months compared to 18 months of patients without prior adjuvant chemotherapy treatment. The influence showed no statistical significance (log-rank test, *p* = 0.80).

Next, we concentrated on patients with adjuvant endocrine resistance. Of *n =* 283 patients with secondary metastatic disease, *n =* 119 patients (42.0%) experienced adjuvant primary endocrine resistance (defined as disease recurrence within the first 2 years of adjuvant ET), and *n =* 61 patients (21.6%) displayed adjuvant secondary endocrine resistance (defined as disease recurrence under adjuvant ET arising after the first 2 years or within 12 months after completion of adjuvant ET; regarding a period of 6 years). For Kaplan-Meier analysis, patients with secondary metastatic disease were divided into 3 groups: (1) adjuvant primary endocrine resistance, (2) adjuvant secondary endocrine resistance, and (3) no endocrine resistance (disease relapse later than 6 years after initial diagnosis). Patients with adjuvant primary endocrine resistance had a statistically significant lower PFS (13 months) compared to patients without adjuvant endocrine resistance (19 months) (log-rank test: *p* = 0.034). Patients with adjuvant secondary endocrine resistance had a median PFS of 18 months under CDK4/6i therapy, without reaching statistical significance when compared to the other two groups (*p* = 0.481 and *p* = 0.312).

**Fig. 4:**
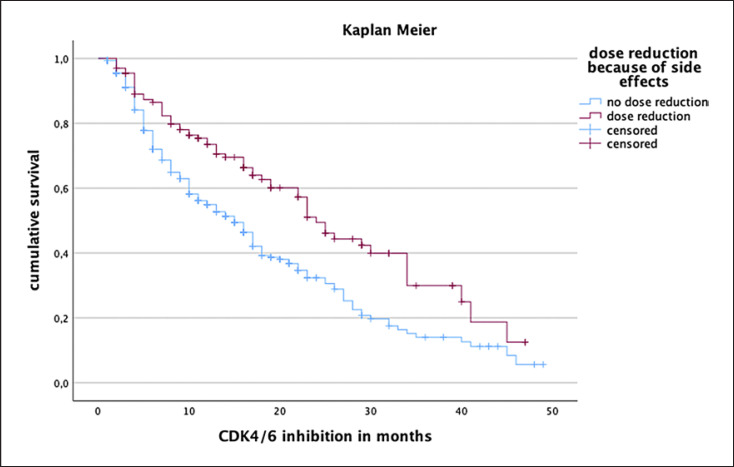
Patients receiving a dose reduction of CDK4/6i had a significantly longer PFS (24 months) compared to patients receiving the recommended dose (15 months). Log-rank test: *p* < 0.001.

Patients, who required a dose reduction of CDK4/6i due to toxicity, had a significantly longer PFS (24 months) compared to patients who received the recommended dosage (15 months; log-rank test: *p* < 0.001; see Fig. 4). To rule out errors, all univariate significant factors influencing PFS were examined by applying multivariate Cox regression analysis with the following factors showing independent statistical significance: patients with liver metastases showed a twice as high risk for earlier disease progression (*p* < 0.001; Hazard ratio 2.036). Further, patients displaying low ER positivity were at a higher risk of earlier disease progression (*p* = 0.004; hazard ratio 1.697), and the therapy line was an independent marker for unfavorable outcomes. With each prior therapy line, the risk of progression increases by 1.57-fold (*p* = 0.02; hazard ratio 1.567). Interestingly, patients who received dose reductions due to toxicity had a 50% risk reduction with respect to disease progression (*p* < 0.001; hazard ratio 0.479). In multivariate regression analysis, a significant influence of lung metastases, progesterone positivity, and Ki67 index could not be proven.

## Discussion

Our study provides RWD on CDK4/6i therapy for HR+/HER2− ABC in Germany over a 4-year period since the approval of palbociclib in November 2016. A particular focus was assigned to clinical outcomes in different lines of therapy, as well as to pre- and post-CDK4/6i treatment approaches. Although all three available CDK4/6i are considered equivalent in the German treatment guidelines [12, 13], patients in the cohort described here were treated predominantly with palbociclib (71.3%). One possible explanation for this preference certainly is the earlier approval of palbociclib prior to the other two CDK4/6i (ribociclib 08/2,017, abemaciclib 09/2,018, respectively). Interestingly, based on the data from the MONALEESA-7 trial [14], the latest German treatment guidelines of the AGO from 2021 favor ribociclib for premenopausal patients in case an aromatase inhibitor is chosen as the endocrine combination partner [12]. This is not yet reflected in our RWD analysis, as the observation period ended prior to the publication of this recommendation. However, based on this guideline, the proportion of premenopausal women treated with ribociclib is expected to increase in the future. Furthermore, recent data from the PALOMA-2 trial failed to show an overall survival benefit for palbociclib + ET compared to ET monotherapy [15], whereas in the MONALEESA-2 trial, ribociclib + ET demonstrated a significant overall survival benefit compared to ET monotherapy [16]. Survival data on abemaciclib are still pending. This might also lead to a switch in CDK4/6i preferences in the future.

**Fig. 5:**
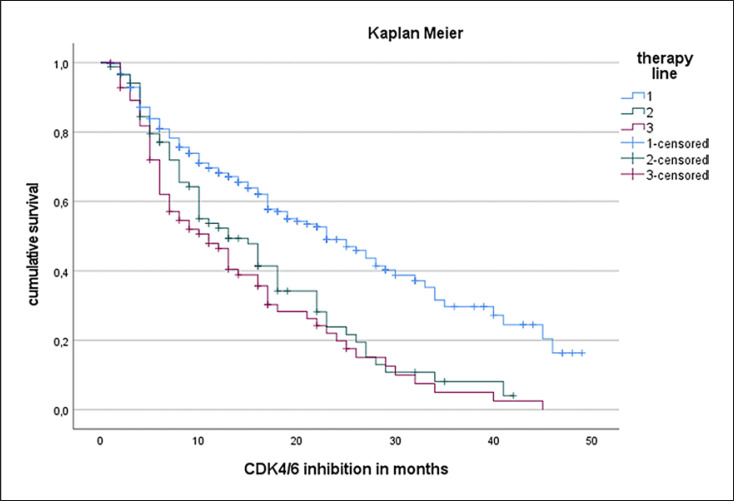
Influence of therapy line in which CDK4/6 inhibitor treatment was administered on median PFS. Group 1: first-line treatment. Group 2: second-line treatment. Group 3: third- or further-line treatment. Median PFS of group 1 was 23 months, group 2 13 months, and group 3 11 months, respectively. Log-rank test *p* < 0.001 (group 1 compared to group 2 and 3).

The most significant difference compared to the pivotal trials is the inclusion of heavily pretreated patients. As shown in Figure 5, PFS differed depending on in which therapy line CDK4/6i was administered. As expected, the more extensive the prior therapy, the shorter the PFS. Excluding patients with higher therapy lines from our analysis, PFS was 23 months (first-line treatment) in the real-world setting. Although the PFS of these patients was significantly higher, it was still slightly lower compared to the pivotal trials (RWD first-line PFS 23 months; PALOMA-2 palbociclib first-line PFS 27.6 months [17]; MONALEESA-2 ribociclib 25.3 months [18]). However, this finding is in line with the literature. A systematic comparison of treatment effects in 21 RCTs and the corresponding real-world datasets demonstrated a 16% lower PFS-rate in the RWD than in the RCT, respectively [19]. In particular, the administration of CDK4/6i in further therapy lines contributed to the shorter median PFS observed within this study compared to pivotal RCTs. In this respect, our data on PFS should be considered as reasonable to expect. This is in-line with a data analysis from the German prospective data registry (“PRAEGNANT”) showing that PFS decreased in further therapy lines of CDK4/6i (CDK4/6i in first-line 24.7 months, second-line 7.8 months, third-line 4.2 months, respectively) [20].

In our data analysis, patients who have previously been treated with chemotherapy in the adjuvant and metastatic setting exhibited a slightly shorter PFS compared to chemonaive patients. This is in-line with data published by Lobezzo et al. [21] and is not surprising as all patients who have received chemotherapy in the metastatic setting were included (also patients who received several previous therapy lines). In addition, patients receiving chemotherapy instead of an endocrine-based therapy in the metastatic setting often belong to a “risk collective” (e.g., impending organ failure or aggressive tumor with high Ki67 index, etc.).

Furthermore, we noticed that dose reduction due to toxicity led to improve PFS during CDK4/6i therapy. It is already known that dose reduction of CDK4/6i due to adverse events does not negatively impact clinical outcomes [22]. In our study, we observed improved outcomes in case of CDK4/6i dose reduction that was initiated due to toxicity. A possible explanation might be that patients with adjusted reduced dosage need fewer therapy interruptions leading to a constant plasma drug level.

We observed that patients with secondary metastatic disease displayed a shortened PFS following CDK4/6i therapy than those who had de novo metastatic disease. Again, this is in line with the literature as patients with secondary metastatic disease have overall worse clinical outcomes compared to de novo metastatic disease [23]. However, within our cohort, this difference was not statistically significant (*p* = 0.21).

Endocrine resistance is playing an important role in HR+/HER2− ABC as approximately half of all HR + patients develop endocrine resistance during treatment [24]. In our study cohort, a total of *n* = 48 of patients with metastatic cancer suffered from primary endocrine resistance. Endocrine resistance in the metastatic situation was defined as disease progression within the first 6 months of first-line CDK4/6i treatment for metastatic BC. Of note, for *n* = 23 (5.1%) of these patients, no tumor biopsy with subsequent receptor determination was performed in the metastatic disease setting. In these patients, accounting for about half of the endocrine-resistant subgroup, endocrine resistance might be due to an unrecognized receptor discordance to HR negative BC. Receptor switch between primary tumor and metastatic disease is of highest relevance in the treatment of patients with metastatic BC. Research has shown that a receptor switch of the ER can occur in approximately 20% of secondary metastatic breast cancer [25]. Hence, the 4th ESO-ESMO International Consensus Guidelines for Advanced Breast Cancer (ABC 4) recommend a biopsy and repeated histological analysis with a consensus of 98% [26]. Of note, endocrine resistance in the adjuvant therapy setting also had a significant impact on PFS following CDK4/6i therapy initiation for treatment of ABC.

In line with previous reports, we further demonstrated that higher ER expression levels were associated with improved PFS. Similarly, patients with progesterone negativity showed decreased PFS [27, 28, 29]. In addition, a high proliferation rate (Ki67 index) in metastases negatively affected PFS, which is also consistent with previous studies [30]. However, correlation of progesterone negativity and proliferation index with PFS did not reach statistical significance in multivariate analyses.

Overall, the administration of CDK4/6i was well tolerated in the study cohort with uncomplicated neutropenia being the most common adverse event. Interestingly, no event of febrile neutropenia was documented in our dataset. With only a small number of patients being treated with abemaciclib, diarrhea, was rare in the current RWD analysis.

Main limitations of our study are the retrospective nature of data collection and the limited cohort size. However, due to an unfiltered inclusion, except for participation in the described pivotal studies, our RWD analysis provides an important overview of the current state of therapy of HR+/HER2− ABC regarding CDK4/6i therapy in Germany. In line with the results from RCTs, our data demonstrated that CDK4/6i is an effective and safe treatment for patients with HR+/HER2− ABC. As inclusion period and data collection ended at the same time in December 2020, follow-up times of patients vary considerably. Therefore, based on our data, it is not feasible to determine data on overall survival. Due to limited cohort size of patients treated with ribociclib and abemaciclib (25.4% and 3.3%, respectively), we refrained from comparing different CDK4/6i among each other. Future research on RWD with longer follow-up is needed to further evaluate the use of CDK4/6i and compare medications within the CDK4/6i class.

## Conclusion

Our RWD analysis on CDK4/6i treatment for patients with HR+/HER2− ABC in Germany indicates treatment efficacy and safety of CDK4/6i. In comparison to data from the approval relevant RCTs, PFS was slightly lower, but within the expected range for RWD which results from inclusion of patients with higher therapy lines into our dataset.

## Statement of Ethics

This research study was conducted retrospectively from data obtained for clinical purposes. All procedures performed in the study involving human participants were in accordance with the ethical standards of the institutional and/or national research committee and with the 1,964 Helsinki declaration and its later amendments or comparable ethical standards. Trial registration numbers were as follows: 35/20 (Ethics Committee of the Saarland Physicians' chamber), 317/21 (Ethics Committee of the Bonn University Hospital), and B302/21 (Ethics Committee of the Christian-Albrechts-University Kiel). This study was supported by Novartis Pharma GmbH as part of the “ERIC” (“Excellent Researchers in Breast Cancer”) project. This project is intended to enable young medical professionals to start their own research projects on breast cancer and was initiated by Novartis Pharma GmbH. Novartis supports externally initiated (e.g., by doctors, scientists, clinics, other institutions), medically/scientifically based, independent research in order to improve the level of knowledge of diseases in therapeutic areas of Novartis' interest.

## Funding Sources

This study was supported by Novartis Pharma GmbH as part of the “ERIC” (“Excellent Researchers in Breast Cancer”) project. See also conflicts of interest section.

## Author Contribution

All authors contributed to the study conception and design. Material preparation and data collection were performed by CM, VK, DJR, and ACR. Data analysis was performed by CM and GW. The manuscript was written by CM, DJR, and ACR. All authors commented on previous versions of the manuscript. All authors read and approved the final version of the manuscript.

## Data Availability Statement

The datasets generated and/or analyzed during the current study are available from the corresponding author on reasonable request.

## Figures and Tables

**Table 1:** Tumor characteristics

	Tumor characteristics			
	tumor characteristics of the primary tumor in secondary metastatic patients		tumor characteristics at metastatic sites	
	*N*	%	*N*	%
Tumor stage (TNM classification)				
pT				
1	94	37.2	−	−
2	109	43.1	−	−
3	34	13.4	−	−
4	15	5.9	−	−
Is	1	0.4	−	−
Total	253	100	−	−
pN				
0	87	33.6	−	−
1	99	38.2	−	−
2	41	15.8	−	−
3	28	10.8	−	−
Positive	4	1.5	−	−
Total	259	100	−	−
Tumor biology				
ER				
Positive	235	83.0	379	84.6
Negative	7	2.5	0	0
X	41	14.5	69	15.4
Total	283	100	448	100
Progesterone receptor (PR)				
Positive	207	73.1	279	62.3
Negative	31	11.0	99	22.1
X	45	15.9	70	15.6
Total	283	100	448	100
Her2				
Positive	9	3.2	0	0
Negative	242	85.5	372	83
X	32	11.3	76	17
Total	283	100	448	100
Grading				
G1	11	3.9	11	2.5
G2	140	49.5	127	28.3
G3	59	20.8	57	12.7
X	73	25.8	253	56.5
Total	283	100	448	100
Ki67				
Mean Ki67	27 (±18)	−	24 (±15)	−

“*X*” = missing data. ER, estrogen receptor.

**Table 2:** Therapy line in which CDK4/6i treatment was administered

Therapy line	Patients, *n*	%
1	278	62.1
2	86	19.2
3	41	9.2
4	18	4.0
5	7	1.6
6	8	1.8
7	5	1.1
8	3	0.6
9	2	0.4

Total	448	100

**Table 3:** Overview of all side effects and differentiation of side effects leading to dose reduction or discontinuation of treatment (*n*) = number of patients; (%) = percentage

Side effects	Side effects (in total)		Dose reduction		Discontinuation of treatment	
	*n*	%	*n*	%	*n*	%
Hematotoxicity (including neutropenia, thrombopenia, pancytopenia)	100	22.3	86	19.2	14	3.1
General condition deterioration (nausea, dizziness, tiredness, etc.)	24	5.4	11	2.5	13	2.9
Treatment initiation with lower dose due to concomitant medication	10	2.2	10	2.2	−	−
Nephrotoxicity	10	2.2	5	1.1	5	1.1
Hepatotoxicity	10	2.2	5	1.1	5	1.1
Rash	11	2.5	4	0.9	7	1.6
Cardiac side effects (including QT time extension)	8	1.8	3	0.7	5	1.1
Dyspnea, cough	3	0.7	3	0.7	−	−
Diarrhea	4	0.9	2	0.4	2	0.4
Others	9	2.0	1	0.2	8	1.8

**Table 4:** Localization of disease progression following CDK4/6i treatment

Progress	Patients, *n*	%
Hepatic	82	18.3
Osseous	31	6.9
Pulmonary	29	6.5
Peritoneal	17	3.8
Pleural	14	3.1
Lymphatic	10	2.2
Cerebral (including meningeal and orbital)	16	3.6

**Table 5:** Overview of previous therapies in metastatic situation, previous therapies in initial disease (in patients with secondary metastasis), and subsequent therapies

	Total, *n* (%)	ET, *n* (%)	Chemotherapy, *n* (%)	Both (endocrine therapy and chemotherapy), *n* (%)
Previous therapy (metastasis)	169 (37.7)	93 (20.8)	20 (4.5)	56 (12.5)
Previous therapy (initial)	268 (59.8)	72 (16.1)	21 (4.7)	175 (39.1)
Subsequent therapy	204 (45.5)	80 (179)	78 (17.4)	46 (10.3)

ET, endocrine therapy.
